# Observations on the Articulation of Artificial Teeth

**Published:** 1877-12

**Authors:** Ad. Hartung


### ARTICLE VII.

### *Observations on the Articulation of Artificial Teeth.*

BY DR. AD. HARTUNG.

The most important point, in applying artificial teeth, be it a partial or a complete substitution, is to obtain a natural meeting of the superior and inferior teeth. It may in some cases be easy to accomplish this; in many others it is accompanied with great difficulty. It is easiest in cases where, by partial loss, the teeth adjacent to the gaps and those opposite have still their straight position, right length, &c., as then the position of the artificial teeth to be made is clearly known, and the biting action follows naturally. On the other hand, the more the remaining natural teeth, single or in clusters, have lost their regularity on account of the remote date of their loss, the more difficult the restoration of a natural biting action will be found. In this case all possible attention is required to supply the requisite of an artificial set, i. e. the restoration of the masticating functions. When an important difference exists between the various points of contact of both the mandibles, the execution is often above the capability of the average technical dentist. We observe in these cases the greatest blunders against a rational contemplation of the indicated relations. We see, for instance, in the case of the insertion of an artificial piece in the upper row, the still existing inferior incisors only strike with the forepart of their edges—or, it may even be, with the upper part of their front sides, upon the rubber, which forms an inclined plane; exactly as if, with the greatest exertion, the inferior teeth should be pushed backward, or, should they resist, the artificial teeth be forced forward.

A very striking proof of such a mistake I observed in a case about two years ago, in which the superior front teeth had been substituted. The artificial piece was only supported by a small natural grinder, while an existing favorable opportunity to get a contact with the lower jaw, further back, had been entirely overlooked. The consequence was that the artificial teeth now extended far beyond the inferior row, and, on account of their forward-inclined position, presented a particularly disagreeable aspect. With their sharp edges at the top they had pushed up the gum of the still remaining roots, and crowns of the opposite natural teeth were inclined forward.

Besides the enumerated disadvantages, the improper treatment throws obstacles in the way of the application of a new and better artificial piece; for instance, when the roots are still solid and the patient for different reasons refuses to have them extracted, and when the gum is loosened at those roots by the sharp edges at the top of the artificial teeth, making the protruberance of the alveoli very prominent.

In another case an artificial piece, of four superior incisors (the rest of the mouth being entirely toothless) had been driven more and more towards the tongue, so as to bring them almost horizontal in the mouth, and consequently the edges of the artificial teeth struck the front side of the inferior teeth, or at the edge of the gum.

The patient had tried in several places to get a set fit for use, but, in cosmetic respect as well as in regard to mastication, she failed, as she was not willing, under any circumstances, to have the four inferior teeth extracted, which, notwithstanding their abnormal position, were quite solid. The request which she now made me, to supply her with a set, inclined me to consider the case in a light entirely different from the view taken heretofore. My opinion was that too much consideration had been given to those still existing teeth. To include them in the row of the new set would always be a failure; therefore I treated the case as if these four teeth had not been in existence, and made a complete inferior set, so that a double row was formed where the four natural teeth remained. The articulation was produced in the usual way. Not to force the natural teeth still further away, the inferior artificial piece was worked in such a manner that in putting it in they slide through a corresponding opening in the hind centre part of it, and in this way rested on a rubber base, while a support for the set was gained at the same time. I felt myself justified in treating the case in the way described, as the patient assured me that the teeth in their abnormal position did not trouble her in any respect, and that the mouth was otherwise entirely in its normal condition. The appearance was very good, and mastication was exceedingly well performed.

After a few years I saw the patient again; the set was still used by her with the same success, and the four inferior teeth remained.

When the jaws are still provided with a few single teeth without any opposite, they are usually very much out of order as to direction and height. Although in this case a good biting action can be produced, yet often the construction of artificial sets is very faulty. In one case an artificial upper piece was broken twice in succession by the solid natural lateral inferior teeth, because they struck the lateral parts of the palate plate only with the outside of their crowns then slid upwards, and working, as it were, knee-shaped forced the plate apart. Had the plate been stronger, and able to longer resist the described influence, the prejudicial consequences would soon have manifested themselves at the respective natural teeth—they would have yielded toward the inside.

By such improper work the operator usually loses sight of the hypothesis that the teeth have to strike each other in such a way *that the direction of the strength in masticating is that of the axis, lengthwise through the teeth*, and should not in the slightest degree cross the axis. This rule is just as well to be observed when the point to be struck is an artificial crown, as when rubber is used. In both cases the surface of the crown has to strike as fully as possible. Therefore I take the wax mould rather broad, to get the full impression of the crowns. Should, however, a natural tooth have so far deviated from its normal position as to make the foregoing hypothesis not applicable, it should, by a proper arrangement of the artificial piece, at least receive a support at the inclining side, to prevent it from a further deviation.

To avoid the denounced errors in artificial substitution, an accurate articulation is, of course, a point of first importance. To give any further information on this point, and how to attain it, would be superfluous after the discussion at this year's meeting of our Schleswig-Holstein Colleges at Kiel. Only this I want to observe that, to my observation the articulation can generally be accomplished in the most accurate and natural way during a perpendicular position of the body and a corresponding direction of the head. Therefore I only admit, as an exception, the correctness of the remark of Dr. Humm, that the bending backward of the head is needful to obtain a correct biting action—just as well as I know that my observation that I " sometimes, in difficult cases, attained the object by bending the head downward," cannot be taken as a general rule. In these cases I cause the chin, while the mouth is open, to be lowered as far as possible on the breast, and fasten it there. To close the teeth, the head should be moved downward to the lower jaw; then particular care should be taken that the patient, when lifting the head, does not lower the inferior jaw in order not to separate the rows of teeth from each other. In this way I often succeed in getting an accurate articulation and the correct biting action when I have failed in all other ways.—*Dental Office and Laboratory.*

